# Spanish validation of short‐form version of Literacy of Suicide Scale (LOSS) and Stigma of Suicide Scale (SOSS)

**DOI:** 10.1002/brb3.3182

**Published:** 2023-07-31

**Authors:** Fernando Collado, Julia Martínez, Adolfo J. Cangas, Rubén Trigueros

**Affiliations:** ^1^ Andalusian Health Service Andalusia Govern Seville Seville Spain; ^2^ Department of Psychology, Health Research Centre University of Almería Almería Spain

**Keywords:** ideation, literacy, prevention, self‐injury, stigma, suicide

## Abstract

**Introduction:**

The evidence shows that a greater understanding of mental health and the myths about suicide is related to a reduction in these types of problems.

**Objective:**

Validate the Literacy of Suicide Scale—Short Form (LOSS‐SF) and the Stigma of Suicide Scale—Short Form (SOSS‐SF) for Spain.

**Materials and methods:**

Both scales were translated to Spanish via backward translation. The sample was comprised of a total of 466 participants, of which 169 (36.2%) were men and 297 (63.6%) were women through nonrandom circumstantial sampling. The questionnaires were administered online. To test the factorial structure and evaluate reliability, both an EFA and an AFC were conducted. In addition, the reliability of the questionnaires was analyzed using the Omega coefficient and Cronbach's alpha. Finally, a linear regression analysis was carried out with the aim of testing the predictability of the scales.

**Results and discussion:**

The results obtained reveal acceptable fit indices and a factorial structure similar to that of the validation conducted for both instruments in other countries. The validity of these instruments for use in Spain is discussed.

## Graphical abstract

Evidence shows that an increased understanding of mental health and suicide myths is related to a reduction of suicide problems. Therefore, validating the Suicide Awareness Scale ‐ Short Form (LOSS‐SF) and the Suicide Stigma Scale ‐ Short Form (SOSS‐SF) is of utmost necessity to have a tool available to detect such cases.

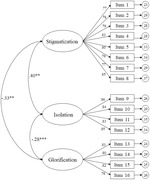

## 1 INTRODUCTION

**FIGURE 1:**
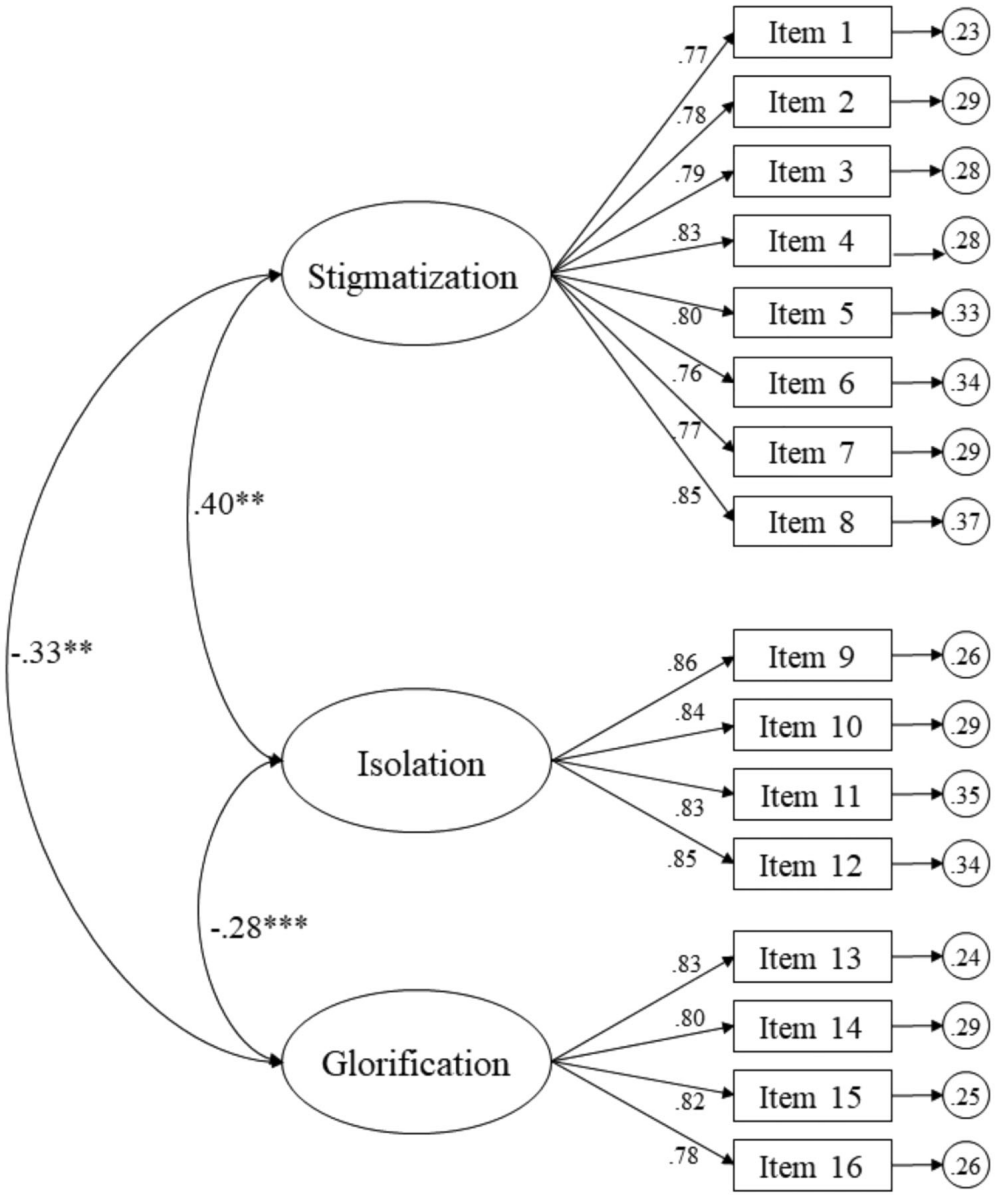
CFA of SOSS‐SF. The ellipses represent the factors and the rectangles represent the different items. The residual variances are shown in the small circles. The sentence corresponding to the item can be found in Appendix B.

According to the World Health Organization, more than 800,000 people commit suicide each year (Castillo, 2022). Suicide attempts, which are not included in this Figure 1, register an even higher number (Aldalaykeh et al., 2020). In Spain, in 2019, 3671 people committed suicide and in 2020 the number of suicides increased to 3941 (De la Torre‐Luque et al., 2022). Based on these data, suicide is confirmed as the number one cause of external death in Spain, doubling deaths caused by traffic accidents (Prieto et al., 2021).

In the past decade, there has been an alarming increase in the suicide rate among young people. According to the most recent data available from the National Statistics Institute (INE in Spanish), in 2018, suicide was the absolute first cause of death among men between 15 and 29 years old and the second among women in the same age group (Prieto et al., 2021). Cases of suicide among young people and middle‐aged adults carry particularly serious connotations given the considerable number of years of life lost and the immense repercussions on the families and those closest to the victims (Aiartzaguena & Morentin, 2022).

In Spain, most psychological autopsies reveal that up to 90% of victims of suicide had one or two psychiatric disorders, specifically depression (Prieto et al., 2021). In this regard, we highlight the research carried out by Aiartzaguena and Morentin (2022) focused on young people (14–34 years old) and middle‐aged adults (35–55 years old). They found that mood disorders were the main risk factor (59%), followed by possessing some physical illness or chronic pain (23%), suicide attempts (20.5%), substance abuse disorders (20.5%), and finally, psychotic disorders (14%). Therefore, the presence of psychiatric disorders is the main risk factor for suicide (Arafat et al., 2022; Santurtún et al., 2018).

Psychiatric and psychological attention is an important strategy in suicide prevention. However, there are important factors that prevent individuals from seeking help, such as the perceived stigma toward suicidal behavior or possessing incorrect information about suicide or mental health (Arafat et al., 2022). Nevertheless, there is evidence which helps to design psychoeducational interventions that reduce stigma and increase general knowledge about suicide (Soto‐Sanz et al., 2019).

Stigma toward suicide refers to a social process that carries overly generalized perceptions, prejudices, and negative attitudes, such as rejection, toward individuals that display suicidal tendencies and people in mourning due to suicide (Wu et al., 2021). The evidence reveals that a greater understanding of mental health in general favors seeking help (Aldalaykeh et al., 2020; Kennedy et al., 2018). Also, it is necessary to evaluate stigma toward suicide within each community to find solutions which aid in combatting it (Aldalaykeh et al., 2020).

In terms of evaluating stigma toward suicide, two of the most widely used instruments in different international studies are the Stigma of Suicide Scale—Short Form (SOSS‐SF), developed by Battherham et al. (2013) and frequently applied in the community (Aldalaykeh et al., 2020) and the Literacy of Suicide Scale—Short‐Form (LOSS‐SF), developed by Calear et al. (2012).

As for the first, SOSS measures the stigma associated with suicide that an individual displays (Arafat et al., 2022). The SOSS consists of three dimensions: an 8‐item factor assessing stigma toward people who die by suicide, a second 4‐item factor attributing suicide to isolation or depression, and a third 4‐item factor on normalization or glorification (Batterham et al., 2013). Regarding LOSS, it evaluates the level of knowledge an individual possesses on the subject of suicide. This scale measures four domains, such as causes that lead to suicide, risk factors, signs or symptoms, and treatment. SOSS and LOSS have been linked to help‐seeking but may also be related to suicide risk reduction as well as suicide prevention (Kennedy et al., 2018).

Both instruments are short and easy to administer. Furthermore, empirical evidence substantiates their possession of commendable reliability and validity, thereby rendering them efficacious measurement instruments deserving of recognition. They have been translated into different languages and validated for Australia, Jordan, Bangladesh, China, and Puerto Rico. Given its application in different countries, in some cases, it has been necessary to modify some items to adjust them to their culture. For example, Arafat et al. (2022) in the SOSS scale linguistically modified item 2, eliminating “psychiatrist” for “mental health professional” due to the inadequate number of psychiatrists in Bangladesh, item 4, given that alcoholism does not represent a public health problem in the Muslim population, and modified the score of item 11 due to the inverted gender pattern in suicide issues in the country. However, despite their application and validation in different cultural settings, both scales have shown high correlations with the original versions, as well as strong factor structures, good internal consistency and convergent validity in all studies. Arafat et al. (2022) argue that it is appropriate to validate scales in different cultural settings because it broadens the applicability of these instruments to diverse populations and contexts. But, they have yet to be validated for Spain (Aldalaykeh et al., 2020; Arafat et al., 2022). For this reason, the present study aims to validate both instruments and use them in Spain.

## 2 METHOD

### 2.1 Participants

The sample was recruited by nonrandom circumstantial sampling, where no inclusion criteria were imposed in order to achieve the broadest possible sample. As exclusion criteria, participants under 18 years of age were excluded.

The sample was comprised of a total of 466 participants, of whom 169 (36.2%) were men and 297 (63.6%) were women. The average age was 46.62 years old (typical deviation 9.82). Most of the participants had university studies (73.4%), 19.3% had completed baccalaureate education, 6% had completed Compulsory Secondary Education, and 1.3% had primary school studies. All participants resided in Spain.

### 2.2 Instruments

#### 2.2.1 The Stigma of Suicide Scale—Short Form (SOSS‐SF) created by Batterham et al. (2012)

The original scale features 58 items and its short form has 16 (Arafat et al., 2022; Batterham et al., 2013). Each item corresponds to an adjective that describes a person who dies as a result of suicide. The participant responds on a Likert scale between “totally disagree” (1) and “totally agree” (5) (Batterham et al., 2013). It has a trifactorial structure in which the key factor evaluates stigma toward people who die due to suicide, another factor addresses the attribution of suicide to isolation or depression, and the last factor assesses normalization or glorification (Batterham et al., 2013). The stigmatization subscale features eight items, that of isolation/depression features four items and normalization/glorification also has four items.

The score of each subscale is calculated by obtaining the average of the responses to the items that represent each subscale. An average score over three shows agreement with the objective concept. For example, the highest scores of the isolation subscale mean more agreement with the idea that attempted suicide is caused by the social isolation or depression of the suicidal person (Aldalaykeh et al., 2020).

The internal consistency of the subscales, measured by Cronbach's alpha, proves suitable, as it is above .78 (Arafat et al., 2022). The original version of the scale also displays very good reliability, registering .86 (Batterham et al., 2013).

#### 2.2.2 The Literacy of Suicide Scale (LOSS) created by Calear et al. (2012)

This scale evaluates the knowledge an individual possesses about suicide based on four dimensions: sign and symptoms (three items), nature or cause of suicide (four items), risk factors (three items), and treatment and prevention (two items) (Aldalaykeh et al., 2020). To evaluate this knowledge, the scale presents a series of statements about suicide, and the person must respond whether each statement is true or false. The original scale features 26 items and the short form has 12. The score is calculated based on the correct answer, meaning the total score ranging between 0 and 12 points (Arafat et al., 2022). Subsequently, this score becomes a percentage, which can range between 0% and 100% (Aldalaykeh et al., 2020).

#### 2.2.3 Sociodemographic characteristics

The instrument developed ad hoc for this study enquires about sex, age, residence, and education level. Additionally, there is a series of brief questions related to suicide and mental health: suicide attempts among family, friends, or the respondent themselves, suicide by a family member or friend, seeking professional psychiatric or psychological help, and the existence of a chronic illness.

### 2.3 Procedure

The first stage of the study involved translating both scales to Spanish. For this purpose, we followed the direct and backward translation procedure established by Hambleton (1996). In this way, a group of translators with experience in the teaching field translated the initial items to Spanish in order that another group of translators could translate those same items from Spanish back to English. Later, the final English version was compared to the original questionnaire. Once the questionnaire in Spanish was obtained, a group of clinical psychologists and psychiatrists modified the items to adapt them to the context of Spain.

The survey was disseminated via social networks and administered online through Google Forms. The objective of the survey was explained to the participants, and they were provided with a contact email address. Moreover, the anonymity of their responses was emphasized with the aim of achieving open and meaningful participation.

The study was approved by the Bioethics Committee of the University of Almeria (Ref. 14/2019 UALBIO), respecting at all times the protocols established in the Helsinki Declaration and the American Psychology Association.

### 2.4 Data analysis

The statistical programs used to test the factorial structure and determine the reliability of SOSS‐SF were SPSS 25 and AMOS 21. Thus, a CFA was carried for the SOSS‐SF. In the case of LOSS, the statistical program SPSS 25 was utilized to conduct an EFA. Subsequently, the reliability of the questionnaires was analyzed via the Omega coefficient and Cronbach's alpha. Finally, a linear regression analysis was conducted with the aim of testing the predictability of the scale and ANOVA in relation to sociodemographic characteristics of the participants.

The maximum likelihood estimation method was used for the CFA, as it is the most suitable when dealing with Likert type questionnaires. Furthermore, the bootstrapping procedure was utilized (6000 interactions). The estimators were not affected by the lack of normality, which is why they were considered robust (Byrne, 2013). To either accept or reject the factorial structure of the subscales, the following fit indices were utilized according to the parameters established by Hair et al. (2006): The incremental indices (CFI, IFI, and TLI) display good fit as long as the score is over .95; RMSEA and SRMR display good fit as long as the score is equal to or below .06; and χ^2^ */gl* displays good fit as long as the score is between 2 and 3.

## 3 RESULTS

### 3.1 Exploratory factorial analysis

The results obtained in the Kaiser–Meyer–Olkin test (KMO = .92) and the Bartlett statistics (χ^2^ (66) = 6532, *p* < .001)) reveal suitable fit indices for LOSS. Also, Table 1 displays the results obtained from the exploratory factorial analysis.

**TABLE 1:** Exploratory factorial analysis LOSS.

Item	Signs and symptoms	Causes/nature of suicide	Risk factors	Treatment and prevention
1	.76	.12	.21	.19
2	.75	.15	.17	−.09
3	.77	.14	−.08	.22
4	.22	.74	.31	.12
5	.16	.78	.20	.29
6	.24	.77	−.07	.28
7	.16	.73	.13	.29
8	−.09	−.36	.74	.22
9	.36	.12	.72	−.07
10	.18	−.08	.79	.20
11	.20	.11	.22	.76
12	.12	−.36	.25	.74

*Note*: The sentence corresponding to the item can be found in Appendix A.

### 3.2 Confirmatory factorial analysis

Para analizar la estructura factorial del cuestionario se agruparon los items en función de cada uno de los factores, siguiendo los postulados de la escala original. Thus, the fit indices shown in the factorial structure of the subscale for SOSS‐SF revealed the following scores: χ^2^ (101, *N* = 466) = 290.56, *p* = .001; χ^2^/df = 2.88; CFI = .97; TLI = .97; IFI = .97; RMSEA = .051 (IC 90% = .045–.059); SRMR = .038. The standardized regression weights were statistically significant (*p* < .001), ranging between .76 and .86.

### 3.3 Descriptive statistics and bivariate correlations

Table 2 shows the descriptive statistics (average and typical deviation), the reliability analyses with Cronbach's alpha and the Omega index and bivariate correlations.

**TABLE 2:** Descriptive statistics, reliability analysis, bivariate correlations, and time stability analysis.

Factors	*M*	*SD*	*α*	*ω*	1	2	3
1. Stigmatization	3.10	1.01	.84	.83	–	.33^**^	.45^***^
2. Isolation	3.38	1.13	.80	.79		–	−.24^***^
3. Glorification	2.23	1.03	.83	.82			–

*** *p* < .001.

** *p* < .01.

Table 3 displays the response scores of the participants for LOSS. The average score was 1.88 over 12, with a pass rate of 59%. Table 4 also shows that the participants had more difficulty on the items related to signs/symptoms and risk factors of suicide. Most of them (80%) responded correctly to the item of seeing a psychiatrist or psychologist can help prevent someone from committing suicide.

**TABLE 3:** Correct responses to items from the Literacy of Suicide Scale.

Items	Percentage correct
Seeing a psychiatrist or psychologist can help prevent someone from committing suicide	84.3
Not all people who try to commit suicide plan the attempt in advance	77.2
There is a strong relationship between alcoholism and suicide	68.3
People who have suicidal thoughts shouldn't tell other people about them	59.4
Most of the people who commit suicide are psychotic	51.3
Very few people have suicidal thoughts	49.5
A suicidal person will always be suicidal and have suicidal thoughts	47.2
Talking about suicide always increases the risk of suicide	41.2
People who want to commit suicide can change their mind quickly	38.4
If they had been evaluated by a psychiatrist, everyone who committed suicide would have been diagnosed as depressed	34.5
People who talk about suicide rarely commit suicide	32.1
Men are more likely to die of suicide than women	30

**TABLE 4:** Linear regression analysis.

	*F*	*R^2^*	*β*	*t*
	34.29	.38^**^		
1. Stigmatization			−.33	−.37^a^
2. Isolation			−.46	−1.11^**^
3. Glorification			−.39	−.78^a^

** *p* < .01.

^a^ *p* < .05.

### 3.4 Linear regression analysis

Table 4 shows the linear regression analysis in which each one of the factors linked to stigma toward suicide were related to literacy of suicide. This analysis aims to reflect the predictability of the scale, whose results show that stigmatization, isolation and glorification were negatively related to LOSS.

Regarding the characteristics of the participants, 189 (40.5%) had a friend who had attempted to commit suicide. In this same line, 135 participants (28.9%) had had a family member attempt suicide. Furthermore, in response to the question as to whether a family member or friend had committed suicide, 136 (29.1%) answered “yes.” Finally, when the participants were asked if they themselves had attempted to commit suicide, 60 (12.8%) answered that they had.

With respect to seeing a psychologist or psychiatrist to seek help, 194 (41.5%) had done so. Similarly, when asked about the existence of a chronic illness, 394 participants (84.4%) answered “no.”

### 3.5 ANOVA

Table 5 shows significant differences in the LOSS and SOS variables between those subjects who have experienced suicide and/or suicide attempt. In detail, when it comes to the SOS and LOSS, it can be observed that those subjects who have felt the loss by suicide or attempt score higher than those who have not experienced it closely. In addition, those subjects who have high scores on the LOSS are those who seek professional psychiatric or psychological help when they are psychologically unwell.

**TABLE 5:** ANOVA sociodemographic variables.

	Suicide attempts among family		Suicide attempts among friends		Suicide attempts among the respondent themselves		Suicide of a family member or friend		Seeking professional psychiatric or psychological help	
	*F*	*p*	*F*	*p*	*F*	*p*	*F*	*p*	*F*	*p*
Stigma of suicide	12.75	.05	8.50	.11	31.88	.001	7.06	.14	9.51	.42
LOSS	21.68	.001	11.46	.24	24.27	.01	23.43	.001	23.42	.001

## 4 DISCUSSION

The present study focused on validating the short‐form versions of LOSS and SOSS in Spain. A sample of 466 people was used. The results reveal that the majority of participants display a significant level of stigma toward suicide and do not possess sufficient knowledge about the subject.

The stigmatization subscale had the highest pass rate among the three SOSS‐SF scales, which indicates that the Spanish population tends to relate suicide to stigma more than social isolation or glorification. This result does not coincide with the Jordanian study by Aldalaykeh et al. (2020) or with the study by Arafat et al. (2022) in Bangladesh. In both of these studies, it was the isolation subscale, which obtained the highest pass rate. However, it is worth adding that this result is very heterogeneous, as Hernández‐Torres et al. (2021) indicate that the stigmatization subscale was the highest and Batterham et al. (2013) state that in their community sample there was more probability to glorify suicide and less probability to attribute it to isolation.

In the Confirmatory Factorial Analysis, the scores of the three SOSS‐SF subscales proved to be quite homogeneous, ranging between 0.74 and 0.86. Hernández‐Torres et al. (2021) obtained similar results in their sample in Puerto Rico, ranging between 0.58 and 0.85. However, the studies conducted in Jordan and Bangladesh displayed varying scores. Said variability might be explained by the cultural beliefs and the similar religion in these countries (Aldalaykeh et al., 2020; Arafat et al., 2022).

The participants showed a relatively low level of literacy of suicide. A pass rate of 59% was obtained, along with an average score of 1.88 over 12. Aldalaykeh et al. (2020) also show that this same result exists among the Jordanian population, obtaining an average score of 5.63 over 12 and a pass rate of 55%.

The participants had difficulty responding to questions about signs and symptoms of suicide, as well as risk factors. However, they responded correctly concerning seeking help or treatment for prevention. These results are similar to the study by Aldalaykeh et al. (2020). Similarly, these authors indicate that stigma, the lack of knowledge and other sociocultural factors can be related to a low rate of literacy of suicide. Batterham et al. (2013) indicates that their community sample in the Australian population had a very low literacy of suicide in general, reflecting very little knowledge about the causes and nature of suicide, as well as treatment and prevention.

Regarding the Cronbach's reliability of SOSS‐SF, the three subscales show a suitable fit, including some cases, which were higher than other studies. For example, the stigmatization subscale displays a value of 0.84, while in Bangladesh in the study by Arafat et al. (2022), it was 0.76. The glorification subscale shows a more homogeneous result in comparison with the other studies, given that in the study in Puerto Rico by Rivera‐Segarra et al. (2018), it is also 0.83 and in the study by Batterham et al. (2013), it is 0.82.

Linear regression analysis shows a negative and significant relationship between SOSS and LOSS. Therefore, we can affirm that suicide literacy prevents the development of suicide stigma. Thus, an adequate predictive validity of both scales is shown. The dimensions of glorification, stigmatization and isolation help to explain the development of stigma by 38% (R2). On the other hand, the results obtained in this study are in line with the findings of previous studies, where suicide literacy has shown the development of accepting behaviors and less prejudice (Aldalaykeh et al., 2020).

The vastness of the sample qualifies in the medium range compared to other studies that have also validated both scales in other populations. For example, the study conducted by Kennedy et al. (2018) in the rural areas of Australia utilized a sample of 536 people, the Jordanian study by Aldalaykeh et al. (2020) used a sample of 160 participants, and in Puerto Rico Rivera‐Segarra et al. (2018) worked with a sample of 123 subjects. In our case, there was greater female participation: 297 women to 169 men. This characteristic, despite the inherent gender imbalance, is also in line with similar investigations. Aldalaykeh et al. (2020) indicate that 60% of their sample were women, and Kennedy et al. (2018) indicate that 251 were women and only 185 were men. Rivera‐Segarra et al. (2018) used a sample in which 56.1% were women, 43.1% were men, and 0.8% was transgender, stating that this distribution is typical in Puerto Rico, where they are more women than men. The sample features a wide range of ages. This characteristic is in line with the study by Batterham et al. (2013), which established an age range from 18 to over 50.

It has been demonstrated that psychological and/or psychiatric attention is an effective tool for suicide prevention (Celano et al., 2017). However, amidst the existence of a significant level of stigma and a scarce level of knowledge about suicide, it is less likely that an individual will seek help from a mental health professional. Thus, it is necessary to strengthen the general population's understanding of suicide, as well as its causes, risk factors, and other related aspects. In this way, and as shown in the literature, sufficient knowledge will reduce the level of stigma toward suicide and increase the probability that people with seek the help they need.

Although the participants stated that they knew seeking help can prevent and treat suicide, the high level of stigma they possessed could impede this option. Moreover, it was also detected that they could not correctly identify, which were the signs and symptoms of suicide, nor the risk factors, details which we consider to be essential given the current relevance of said phenomenon.

Finally, the limitations of the present work must be mentioned. First, with regard to the administering of the scales, we cannot guarantee that the participants responded to the questionnaires themselves. Second, there may have been some bias in the sample, which may not have been representative of the general population due to the method in which it was accessed. Finally, it was not possible to perform the concurrent validity analysis. For future research, it would be interesting to increase the sample size in order to achieve greater representativeness, to retest the factorial structure of both questionnaires since it is a continuous process, and finally, to perform the concurrent validity analysis with a bilingual population in order to know if both questionnaires are understood in a similar way.

## CONFLICT OF INTEREST STATEMENT

We wish to confirm that there are no known conflicts of interest associated with this publication and there has been no significant financial support for this work that could have influenced its outcome. We confirm that the manuscript has been read and approved by all named authors and that there are no other persons who satisfied the criteria for authorship but are not listed. We further confirm that the order of authors listed in the manuscript has been approved by all of us.

### PEER REVIEW

The peer review history for this article is available at https://publons.com/publon/10.1002/brb3.3182.

## Data Availability

The data that support the findings of this study are available from the corresponding author, upon reasonable request.

## APPENDIX A

### A.1

La escala presenta una serie de afirmaciones sobre el suicidio, y debéis de responder si cada afirmación es verdadera o falsa.

**Table:**

1. Si fueran valorados por un psiquiatra, todos los que se suicidan serían diagnosticados de depresión.
2. Consultar a un psiquiatra o psicólogo puede ayudar a prevenir que alguien se suicide.
3. La mayoría de las personas que se suicidan son psicóticas.
4. Existe una fuerte relación entre el alcoholismo y el suicidio.
5. Las personas que hablan del suicidio rara vez terminan suicidándose.
6. Las personas que quieren intentar suicidarse pueden cambiar de opinión rápidamente.
7. Hablar de suicidio siempre aumenta el riesgo de suicidio.
8. No todas las personas que intentan suicidarse planifican su intento con antelación.
9. Las personas que tienen pensamientos suicidas no deben decírselo a otros.
10. Muy pocas personas tienen pensamientos suicidas.
11. Los hombres son más propensos a suicidarse que las mujeres.
12. Una persona suicida siempre será un suicida y tendrá pensamientos de suicidio.

*Note*: The LOSS was validated in Spanish.

## APPENDIX B

### B.1

En general las personas que mueren por suicidio son…

**Table:**

1. Patéticas
2. Cobardes
3. Superficiales
4. Estúpidas
5. Vengativas
6. Irresponsables
7. Inmorales
8. Una vergüenza
9. Solitarias
10. Perdidas
11. Aisladas
12. Desconectadas
13. Valientes
14. Nobles
15. Fuertes
16. Decididas

*Note*: The SOSS‐SF was validated in Spanish.
